# Race affects SVR12 in a large and ethnically diverse hepatitis C‐infected patient population following treatment with direct‐acting antivirals: Analysis of a single‐center Department of Veterans Affairs cohort

**DOI:** 10.1002/prp2.379

**Published:** 2018-02-22

**Authors:** Jihane N. Benhammou, Tien S. Dong, Folasade P. May, Jenna Kawamoto, Ram Dixit, Samuel Jackson, Vivek Dixit, Debika Bhattacharya, Steven B. Han, Joseph R. Pisegna

**Affiliations:** ^1^ Division of Gastroenterology, Hepatology and Parenteral Nutrition Department of Medicine David Geffen School of Medicine at UCLA Los Angeles CA USA; ^2^ Vatche & Tamar Manoukian Division of Digestive Diseases Los Angeles CA USA; ^3^ Division of Infectious Diseases VA Greater Los Angeles Healthcare System Department of Medicine David Geffen School of Medicine at UCLA Los Angeles CA USA

**Keywords:** Adherence, direct‐acting antivirals, direct drug acting, drug metabolism, ethnicity, Hepatitis C, medication procession ratio, polymorphisms, race, racial disparity, sustained virological response 12, Veterans Affairs

## Abstract

Hepatitis C virus (HCV) infection is a major cause of chronic liver disease. HCV cure has been linked to improved patient outcomes. In the era of direct‐acting antivirals (DAAs), HCV cure has become the goal, as defined by sustained virological response 12 weeks (SVR12) after completion of therapy. Historically, African‐Americans have had lower SVR12 rates compared to White people in the interferon era, which had been attributed to the high prevalence of non‐CC interleukin 28B (IL28B) type. Less is known about the association between race/ethnicity and SVR12 in DAA‐treated era. The aim of the study is to evaluate the predictors of SVR12 in a diverse, single‐center Veterans Affairs population. We conducted a retrospective study of patients undergoing HCV therapy with DAAs from 2014 to 2016 at the VA Greater Los Angeles Healthcare System. We performed a multivariable logistic regression analysis to determine predictors of SVR12, adjusting for age, HCV genotype, DAA regimen and duration, human immunodeficiency virus (HIV) status, fibrosis, nonalcoholic fatty liver disease (NAFLD) fibrosis score, homelessness, mental health, and adherence. Our cohort included 1068 patients, out of which 401 (37.5%) were White people and 400 (37.5%) were African‐American. Genotype 1 was the most common genotype (83.9%, N = 896). In the adjusted models, race/ethnicity and the presence of fibrosis were statistically significant predictors of non‐SVR. African‐Americans had 57% lower odds for reaching SVR12 (adj.OR = 0.43, 95% CI = 1.5‐4.1) compared to White people. Advanced fibrosis (adj.OR = 0.40, 95% CI = 0.26‐0.68) was also a significant predictor of non‐SVR. In a single‐center VA population on DAAs, African‐Americans were less likely than White people to reach SVR12 when adjusting for covariates.

**Abbreviations**
BMI: body mass index
CI: confidence interval
CYP: cytochrome P450
DAAs: direct‐acting antivirals
Fib4: fibrosis‐4
HCC: hepatocellular carcinoma
HCV: hepatitis C virus
HIV: human immunodeficiency virus
ICD: international classification of diseases
MPR: medication procession ratio
NAFLD: nonalcoholic fatty liver disease
OR: odds ratio
RNA: ribonucleic acid
SVR12: sustained virological response at 12 weeks

## 1 INTRODUCTION

Hepatitis C remains a burden with at least 5.2 million infected people worldwide.^1^ Long‐term infection can lead to complications including cirrhosis, hepatocellular carcinoma, and death.^2^ Eradication of HCV has become a focus given that sustained virological response (SVR) has been associated with reversal of hepatic fibrosis and decreased rates of HCC.^3^, ^4^

Historically, race has played a major role in chronic hepatitis C rates and treatment responses with African‐Americans being disproportionately affected.^5^, ^6^ Host immune responses largely explained these differences as African‐Americans were more likely to harbor the non‐CC interleukin IL28B genotype which affected their response to pegylated interferon.^7^, ^8^ More recently, new direct‐acting antivirals (DAAs) have revolutionized hepatitis C therapy given their ease of administration, tolerability and reported sustained virological response at 12 weeks (SVR12) with rates in the 90s, depending on the extent of liver fibrosis and genotype.^9^ Despite these strides, racial differences in treatment outcomes remain. In large cohorts of patients with genotype 1 treated with sofosbuvir/ledipasvir with or without ribavirin, Africans‐Americans were shown to have higher rates of relapse, although these differences disappeared after extending therapy from 8 to 12 weeks.^10^ These data were corroborated in a larger Veterans Affairs cohort where African‐American patients treated with 12 weeks of therapy instead of 8 reached SVR12 rates similar to their White counterparts.^11^ More recently, Su and colleagues demonstrated that African‐Americans in the VA treated with DAAs have lower SVR rates than White Veterans. In a multivariable regression model, adjusting for factors that can affect SVR, the adjusted odds ratio for African‐Americans was 0.77 for reaching SVR compared to White people (95% CI = 0.69‐0.87), where 29% of the population was African‐American (N = 6171).^12^ Whether these race‐based differences in SVR, particularly with abbreviated regimens, both in the interferon and DAA eras are due to genetic or other socioeconomic factors remains to be elucidated.

The disproportionate HCV infection rates in African‐Americans and higher risk of developing HCC independently of fibrosis underscores the importance of understanding why these differences exist.^13^, ^14^ The aim of our study is to address if racial/ethnic differences in SVR12 are observed in a large and ethnically diverse single‐center Veterans Affairs cohort treated with DAAs or all genotypes, when controlling for HCV treatments, extent of fibrosis, and treatment adherence.

## 2 MATERIALS AND METHODS

### 2.1 Data source

This was an observational retrospective study of all HCV‐infected patients treated at the VA Greater Los Angeles Healthcare System within the Corporate Data Warehouse for a diagnosis of chronic hepatitis C by the International Classification of Diseases, ICD‐9 or ICD‐10, coding. Data were extracted from January 1, 2014 to December 31, 2016 and included: baseline demographic and clinical characteristics, medication, laboratory results, outpatient visits, and previous diseases/diagnoses.

### 2.2 Study population

The study population consisted of consecutive HCV‐infected patients who received DAA therapy at VA Greater Los Angeles Healthcare System. Patients without SVR data available 12 or more weeks following antiviral therapy were excluded from analysis. All genotypes 1‐6 were included. Choice of DAA regimen was at the discretion of the provider. On‐treatment and posttreatment monitoring followed an established protocol that included serum SVR evaluation every 2‐4 weeks.

### 2.3 Sustained virological response

The primary outcome of our study was SVR12, which was defined as an undetectable HCV RNA (<15 IU/mL) 12 weeks or beyond the conclusion of treatment.^15^

### 2.4 Baseline characteristics

Baseline demographic variables obtained at the initiation of therapy included: age, self‐reported race and ethnicity; HCV genotype; nonalcoholic fatty liver (NAFLD) fibrosis score (<−1.455 being unlikely to have advanced NAFLD fibrosis vs >0.676 being predictive of advanced NAFLD‐associated fibrosis)^16^; the Fibrosis‐4 (Fib4; which will be referred to as advanced fibrosis from here forward), as a marker of advanced liver disease using the formula (age x aspartate aminotransferase)/(platelets X alanine aminotransferase^1/2^)^17^; body mass index (BMI) (≥30 kg/m^2^ and <30 kg/m^2^); HIV status; and HCV treatment status (naïve or experienced). For race and ethnicity, we used a single variable that combines concepts of race and ethnicity into five mutually exclusive categories for race/ethnicity: non‐Hispanic White, non‐Hispanic Black (African‐Americans), Hispanics, Asians, and Unknown/Other. The presence of diabetes and its complications were determined by ICD‐9 (250.00‐250.92) and ICD‐10 codes, as were diagnoses of hypertension, hyperlipidemia, HIV, and AIDS with their complications. Psychiatric disorders, both organic and nonorganic (associated with substance abuse or not) were included in our analysis as was substance abuse and homelessness.

### 2.5 Medication adherence

Patient adherence was assessed by calculating the medication procession ratio (MPR) since this has been a validated method to determine adherence.^18^ MPR was defined by the total number of pills supplied over the total number of pills expected to be dispensed by the pharmacy department based on length of treatment regimen and genotype. For purposes of simplicity, “MPR” will be defined and described as “adherence” from here onward.

### 2.6 Statistical analysis

Demographic data which included sex, race/ethnicity, DAA regimen, body mass index (BMI), advanced fibrosis, and genotype were summarized with frequencies and chi‐square tests for comparisons. We conducted multivariable logistic regression analysis to model predictors of SVR12. A priori covariates selected for the model were age, race/ethnicity, genotype, treatment regimen, treatment length, being treatment naïve, HIV status, advanced fibrosis, NAFLD fibrosis score, and adherence as defined by adherence. Age and adherence were continuous variables in the analysis. To analyze the adherence data further, we stratified adherence into the following groups: ≥90%, 80%‐89%, 60%‐79%, and <60%. In addition to using adherence as a continuous variable we also performed regression models with MRP with the defined groups as a categorical covariate. Prior to regression analyses, we tested for multiple collinearity, and no covariates were collinear as defined as a variance inflation factor of less than 10. Separate logistic multivariable regressions models were used to model SVR predictors in the African‐American subgroup by assessing treatment length of 8‐ versus 12‐week treatment, removing what is now considered suboptimal therapies (sofosbuvir/simeprevir ± ribavirin and sofosbuvir/ribavirin). We also performed regression models by race/ethnicity, genotype, and adherence on SVR12.

This study was approved by the Institutional Review Board and Research and Development Committee at the VA Greater Los Angeles Healthcare System.

## 3 RESULTS

### 3.1 Baseline characteristics and treatment regimens

Of the 1204 patients meeting the inclusion and exclusion criteria, 1068 patients were included for analysis based on having complete demographic and follow‐up data. Baseline characteristics of the cohort are presented in Table 1. Males comprised 97% of the study cohort, consistent with gender demographics within the VA system. The mean age was 61.8 (SE ± 0.2). White people and African‐Americans were equally represented at 37.8% of the population. Genotype 1 (1a and 1b) was the most common genotype in 83.9% of patients (N = 896), followed by genotype 2 (7.9%, N = 84) and genotype 3 (6.9%, N = 74). None of the patients had genotype 5. Of all patients, 35.4% were considered to have advanced liver disease as defined by a Fib4 > 3.25. A minority of patients were HIV positive (3%, N = 35). The majority of patients were treatment naïve at the time of DAA initiation at 79.5% (N = 849).

**Table 1:** Demographics of study population

Demographic	N = 1068
Age in years, mean	61.8
SVR, %	87.0
Race/ethnicity, % (N)	
White people	37.5 (400)
African‐American	37.5 (401)
Hispanic	15.1 (161)
Asian	0.7 (7)
Other/Unknown	21 (9.3)
Genotype 1 (a & b), % (N)	83.9 (896)
Treatment naïve, % (N)	79.5 (849)
BMI > 30, % (N)	33.5 (358)
Advanced fibrosis, % (N)	35.4 (462)
HIV positive, % (N)	3.3 (35)
Homelessness, % (N)	22 (236)
Substance abuse, % (N)	27.9 (298)
History of psychiatric disorder, % (N)	58.5 (625)
Medical procession ration, % (N)	
<60%	5.3% (57)
60‐79%	0.5% (5)
80‐89%	0.2% (2)
≥90%	94% (1004)

DAA treatment regimen allocations for all patients and the corresponding SVR12 for each genotype are summarized in Table 2. The most common regimen was sofosbuvir/ledipasvir ± ribavirin, which occurred for 47.8% of the population. Since the study cohort included patients started on antiviral therapy from January 1, 2014, our data also include older antiviral regimens such as sofosbuvir/simeprevir (17.5%, N = 187), sofosbuvir/simeprevir ± ribavirin (0.7%, N = 7), and sofosbuvir/ribavirin (9.8%, N = 105). Our data also include a subgroup of African‐Americans patients who only received 8 weeks of therapy instead of 12 (N = 159) based on pretreatment viral level. These patients were treated before 2015 and prior to recommendations to use 12‐week regimens in African‐Americans.^10^

**Table 2:** Treatment regimen allocation and SVR12 rates for all patients and genotypes

Treatment regimen by genotype	SVR12, % (N)	
Genotype 1	1a	1b
Sofosbuvir/ledipasvir	95.0 (314)	90.0 (116)
Sofosbuvir/ledipasvir/ribavirin	87.2 (89)	92 (27)
Sofosbuvir/ribavirin	100.0 (1)	85.5 (1)
Sofosbuvir/simeprevir	78.0 (138)	100.0 (57)
Sofosbuvir/simeprevir/ribavirin	100.0 (8)	100.0 (1)
Paritaprevir/ritonavir/ombitasvir + dasabuvir	100.0 (1)	91.5 (47)
Paritaprevir/ritonavir/ombitasvir + dasabuvir/ribavirin	83.6 (152)	91.5 (50)
Grazoprevir/elbasvir	100.0 (1)	100.0 (8)
Genotype 2		
Sofosbuvir/ribavirin	77.8 (97)	
Sofosbuvir/ledipasvir/ribavirin	100.0 (1)	
Genotype 3		
Sofosbuvir/ledipasvir	100.0 (1)	
Sofosbuvir/ledipasvir/ribavirin	82.1 (43)	
Sofosbuvir/ribavirin	61.1 (20)	
sofosbuvir/daclatasvir	83.3 (7)	
Sofosbuvir/daclatasvir/ribavirin	81.8 (13)
Genotype 4		
Sofosbuvir/ledipasvir	100.0 (8)	
Sofosbuvir/ribavirin	100.0 (3)	
Paritaprevir/ritonavir/ombitasvir + dasabuvir/ribavirin	100.0 (1)	
Paritaprevir/ritonavir/ombitasvir/ribavirin	100.0 (2)	
Genotype 6		
Sofosbuvir/ledipasvir	100.0 (1)	

SVR12, sustained virological response at 12 weeks.

### 3.2 Predictors of SVR12

Predictors of SVR12 from adjusted regression models are summarized in Table 3. There were two clinically significant negative predictors of SVR12: African‐American race/ethnicity (aOR = 0.43; 95% CI = 0.27‐0.69) and advanced liver disease (Fib4 score >3.25) (aOR = 0.4; 95% CI = 0.26‐0.68). Covariates that did not affect SVR12 included age, genotype, HIV status, advanced NAFLD fibrosis score, BMI ≥ 30 kg/m^2^, and whether the patient was treatment naïve or experienced.

**Table 3:** Odds ratios for SVR12 for all patients

Patient characteristic	Unadjusted		Adjusted	
	OR	95% CI	OR	95% CI
African‐American (ref. White people)	0.77	0.54‐1.11	0.43	0.27‐0.69
Age	1.02	1.00‐1.05	1.04	1.00‐1.07
Genotype 2 (ref. genotype 1)	0.5	0.29‐0.88	2.47	0.25‐24.5
Treatment Naïve (ref. treatment experienced)	1.44	0.95‐2.17	1.46	0.90‐2.37
Advanced fibrosis (ref. Fib4 < 3.25)	0.4	0.28‐0.58	0.40	0.26‐0.68
BMI ≥ 30 (ref. BMI<30 kg/m^2^)	0.9	0.62‐1.31	1.18	0.79‐1.78
HIV positive (ref. HIV negative)	1.44	0.43‐4.8	1.77	0.48‐6.54
MPR 80‐89% (ref. ≥90%)	0.15	0.01‐2.37	a	a
MPR 60‐79% (ref. ≥90%)	0.04	0.004‐0.32	<0.1	0
MPR <60% (ref. ≥90%)	0.02	0.01‐0.05	0.01	13‐166

a Omitted from the analysis given low number of patients (n=2).

### 3.3 SVR12 by race/ethnicity

SVR12 differences by race/ethnicity were also observed. African‐Americans reached SVR12 85% of the time, while White people had SVR rates of 89% and Hispanics of 83%. When older therapies (sofosbuvir/simeprevir, sofosbuvir/simeprevir ± ribavirin, sofosbuvir/ribavirin) were excluded from the analysis, African‐Americans reached SVR12 87.8% of the time, while White people and Hispanics achieved SVR12 rates of 92.4% and 88.7%, respectively. Hispanics had an adjusted OR of 0.75 (95% CI = 0.43‐1.31); Asians of 0.61 (95% CI = 0.67‐5.43); and other/unknown race/ethnicity of 0.67 (95% CI = 0.13‐3.37).

### 3.4 SVR12 in African‐Americans by treatment duration subgroup

Given previously published data suggesting that African‐American patients require 12 weeks of therapy regardless of baseline viral load, we further investigated SVR12 rates for African‐Americans when stratifying by duration of treatment (8 weeks and 12 weeks).^10^, ^11^ Only 159 African‐American patients were treated for 8 weeks of therapy given the recent change in clinical practice. The adjusted odds ratio for SVR12 for all genotypes in the 8‐week group was 0.34 (95% CI = 0.09‐1.29) compared to 0.4 (95% CI = 0.25‐0.63) in the 12‐week treatment group (N = 1043).

There were 746 African‐American patients who were treated with DAA regimens other than sofosbuvir/ledipasvir ± ribavirin and sofosbuvir/ribavirin. In this subgroup, the adjusted OR for SVR12 among African‐Americans was 0.45 (95% CI = 0.25‐0.81), consistent with a significantly lower SVR12 for those only using optimal therapy when compared to those on obsolete therapies. However, when adherence, as defined by MPR, was included in the model, the adjusted OR was 0.47 (95% CI = 0.21‐1.07), mitigating the effect. When adjusting for homelessness, substance abuse, and mental health disorders (N = 306), only homelessness affected SVR12 rates among African‐Americans (aOR = 0.39; 95% CI = 0.19‐0.81).

### 3.5 SVR12 in genotype 1 patients

Given the heterogeneity of our patient population in genotype and therapies, as well as the observation that genotype 3 is more difficult to eradicate,^19^ we performed subanalyses on patients with only genotype 1 disease (1a and 1b) (N = 872). Similar to the larger cohort, African‐American race/ethnicity was a significant predictor for non‐SVR12 with an adjusted OR of 0.48 (95% CI = 0.29‐0.80). When older therapies were excluded (N = 69), being African‐American race/ethnicity remained a significant predictor with an adjusted OR of 0.47 (95% CI = 0.23‐0.82). When adherence was included in the model, African‐American race/ethnicity was not a significant predictor (aOR = 0.60; 95% CI = 0.31‐1.17). When addressing SVR12 only in the African‐American subgroup treated for genotype 1 (N = 358), the only predictor of SVR12 failure was advanced liver disease (aOR = 0.35; 95% CI = 0.12‐0.97).

## 4 DISCUSSION

This study demonstrates that in a large ethnically diverse community‐based Veterans Affairs practice, SVR12 rates for chronic hepatitis C are influenced by race/ethnicity and advanced liver disease, which corroborates previously published data.^10^, ^12^, ^20^ In our cohort, the lower SVR rates observed in African‐Americans relative to White people is persistent despite at least 12 weeks of therapy even when only using current “optimal” therapies. One important consideration in our analysis was the effect of adherence by measures of MPR in an ethnically diverse population treated with direct‐acting antivirals, which has not been evaluated in detail previously. We find that adherence explains some of these differences. These data suggest potential underlying biological differences between White people and African‐Americans in medication response.

While adherence and medication tolerability are not a concern in well‐resourced large clinical trials, they can be more difficult to measure in real‐world effectiveness data for chronic hepatitis C treatment. In 2007, Backus and colleagues assessed SVR12 rates in a Veterans Affairs cohort during the interferon era and demonstrated adherence, as defined by MPR, to be a predictor of SVR success. Patients with adherence of 90% or greater reached SVR 88% of the time. There was a clear threshold where any adherence less than 80% negatively impacted SVR, resulting in SVR12 rates of only 8%.^20^ More recently in 2017, Louie and colleagues assessed real‐world effectiveness of SVR12 with the use of DAAs in the Kaiser Permanente Southern California health care system. Although African‐Americans only represented 8% of their population (N = 17), adherence was also a predictor of success with an adjusted OR of 2.28 if it reached 80% or higher.^21^ Our study demonstrated similar findings. We find that patients with adherence rates of <90% did not reach SVR12. Adherence appeared to attenuate the association between race/ethnicity and SVR12, thus explaining some of the differences observed, although biological causes have not been addressed.

One potential biological explanation for these observations is differences in drug metabolism, driven by the patients’ genetic background. Drug metabolism differences have been described to exist between African‐Americans and White people. Although some of these differences have been attributed to environmental factors such as diet and concomitant medications, intrinsic host factors such as genetic variability and gene polymorphisms in drug metabolizers such as CYP2D6 and CYP2C19 have also been described.^22^, ^23^ Understanding genetic polymorphisms in African‐Americans and elucidating their mechanism of action, namely in the IL28B gene also offered great advances in understanding the underlying genetic differences between African‐Americans and White people in the interferon era.^7^, ^24^ Other genetic differences such as the variant in HAVCR1 gene variant (rs6880859) were also subsequently identified.^25^ Although the polymorphisms and genetic differences listed above have not been shown to affect SVR12 with DAAs, such undiscovered genetic polymorphisms could explain these findings. The effects of genetic variants on SVR in the DAA era have not been addressed systematically other than to assess patterns of resistance.^26^ The previous findings that extending therapy in African‐Americans from 8 to 12 weeks to reach similar SVR12 to White people, also points to potential biological differences between different races and ethnicities. To address this, Large Genome Wide Association Studies (GWAS) or comparing genotypes in African‐Americans and White people who reached and did not reach SVR are needed.

Another consideration in our analysis is the effect of genotype on each race/ethnicity. Although we did not stratify our data by genotype for each race, it is possible that some of the differences observed were driven by the virus genotype. For example, the aOR for genotype 2 dramatically improved (0.5‐2.5) after adjusting for other covariates. All patients treated with sofosbuvir/ribavirin were considered “sub‐optimal” with SVR12 rates of 77.8%. Of that group, 11 patients were African‐American. To address this further, SVR12 in African‐Americans would have to be evaluated with new and “optimal” therapies.

While our study highlights important differences in DAA responses by race/ethnicity, there are limitations. Given the predominance of males in the VA population, our data may not be generalizable to female patients. Similarly, the small number of HIV‐confected patients in our cohort cannot provide conclusive observations about SVR12 rates in this group. The VA Greater Los Angeles Healthcare System covers a large geographical region within the Los Angeles area. It remains unclear at this time if the lower adherence rates observed in African‐Americans is associated with geographical regions. Our facilities offer telemedicine and remote access care which was taken into account in our adjusted model (data not shown), thus making access to care an unlikely etiology. Another possibility for the large MRP effects in African‐Americans is medication tolerability/side effects, which could also be related to the intrinsic metabolism of these medications. Ideally, metabolite levels would have been checked to further assess this; however, given the retrospective nature of our study, this was not addressed. Examining the true associations between socioeconomic status and adherence can be more challenging and would require a root‐cause analysis.

In conclusion, we present a large, ethnically and medically heterogeneous population within the Veterans Affairs System and their SVRs rates in the DAA era. Although our data demonstrate the importance of racial/ethnic differences, the true etiology of these differences remains unclear, which can be further explored in prospective studies where drug levels and patient genetics are taken into account.

## AUTHOR CONTRIBUTION

JNB, TSD, FPM, and JK helped with the study concept and design; JNB, TSD, JK, SJ, RD, and VD helped in the acquisition of data; JNB, TSD, FPM, and DB analyzed and interpreted the data; JNB, FPM, and DB drafted the manuscript; JNB, TSD, FPM, DB, SH, and JRP helped with the critical revision of the manuscript for important intellectual content; TSD performed the statistical analysis; obtained funding (not applicable); JRP helped with the study supervision.

## DISCLOSURE

None declared.
